# Preference for wine is associated with lower hip fracture incidence in post-menopausal women

**DOI:** 10.1186/1472-6874-13-36

**Published:** 2013-09-22

**Authors:** Jessica T Kubo, Marcia L Stefanick, John Robbins, Jean Wactawski-Wende, Mark R Cullen, Matthew Freiberg, Manisha Desai

**Affiliations:** 1 Quantitative Sciences Unit, Stanford University School of Medicine, 1070 Arastradero Road, Palo Alto, Stanford, CA 94304, USA; 2 Stanford Prevention Research Center, Stanford University School of Medicine, Stanford, CA, USA; 3 Department of Internal Medicine, University of California-Davis, Davis, CA, USA; 4 Department of Social and Preventive Medicine, University at Buffalo, The State University of New York, Buffalo, NY, USA; 5 Department of Medicine, Division of General Medical Disciplines, Stanford University School of Medicine, Stanford, CA, USA; 6 Division of General Internal Medicine and Center for Research on Health Care, University of Pittsburgh, Pittsburgh, PA, USA

**Keywords:** Alcohol, Wine, Hip fracture, Osteoporosis, Women’s Health Initiative

## Abstract

**Background:**

Past studies of relationships between alcohol and hip fracture have generally focused on total alcohol consumed and not type of alcohol. Different types of alcohol consist of varying components which may affect risk of hip fracture differentially. This study seeks to examine the relationship between alcohol consumption, with a focus on type of alcohol consumed (e.g. beer, wine, or hard liquor) and hip fracture risk in post-menopausal women.

**Methods:**

The longitudinal cohort consisted of U.S. post-menopausal women aged 50–79 years enrolled between 1993–1998 in the Women’s Health Initiative Clinical Trials and Observational Study (N=115,655).

**Results:**

Women were categorized as non-drinkers, past drinkers, infrequent drinkers and drinkers by preference of alcohol type (i.e. those who preferred wine, beer, hard liquor, or who had no strong preference). Mean alcohol consumption among current drinkers was 3.3 servings per week; this was similar among those who preferred wine, beer and liquor. After adjustment for potential confounders, alcohol preference was strongly correlated with hip fracture risk (p = 0.0167); in particular, women who preferred wine were at lower risk than non-drinkers (OR=0.78; 95% CI 0.64-0.95), past drinkers (OR=0.85; 95% CI 0.72-1.00), infrequent drinkers (OR=0.73; 95% CI 0.61-0.88), hard liquor drinkers (OR=0.87; 95% CI 0.71-1.06), beer drinkers (OR=0.72; 95% CI 0.55-0.95) and those with no strong preference (OR=0.89; 95% CI 0.89; 95% CI 0.73-1.10).

**Conclusions:**

Preference of alcohol type was associated with hip fracture; women who preferentially consumed wine had a lower risk of hip fracture compared to non-drinkers, past drinkers, and those with other alcohol preferences.

## Background

Hip fractures are a major public health problem worldwide [1], contributing to decreased quality of life and premature death [1-6]. In the United States, over 280,000 people over the age of 65 experienced a hip fracture in 2007 [5]. In 2005, the estimated total cost of hip fractures in the US was $12 billion and was estimated to increase 50% by 2025 [7]. More than two-thirds of all hip fractures occur in women [5,8] and older age significantly increases the risk of fracture [5,9,10] with those 85 and older having a ten-fold risk compared to those who are 60–65 [5]. Other factors known to be associated with hip fracture incidence include low body mass index [11], European or Asian race/ethnicity [12,13], smoking [14,15] and less physical activity [2,13,16]. A previous analysis of Women’s Health Initiative (WHI) Observational Study (OS) data identified each of these factors as important predictors of hip fracture [17].

Light-to-moderate alcohol consumption has been shown to be associated with reduced risk of hip fracture and increased bone density [18-22]. More precisely, a U-shaped relationship has been observed in which non-drinkers and heavy drinkers have an elevated risk of hip fracture compared to light-moderate drinkers. The 2010 Dietary Guidelines for Americans defines moderate alcohol consumption as up to one drink per day for women [23].

While various studies suggest that alcohol consumption may be related to hip fracture, the risk of hip fracture may be different for those who consume beer, wine, and hard liquor as was observed for cardiovascular disease [24,25] and overall mortality [24-28]. In a study of 31,785 men and women in Denmark, Høidrup et al. [19] assessed preference of alcohol type among current alcohol consumers and found that those who preferred wine had a reduced risk of hip fracture compared to those who preferred beer or liquor. In contrast, in their analysis of the Cardiovascular Health Study, Mukamal et al. [20] found that the reported consumption of beer, wine and hard liquor did not have a significant association with hazard of hip fracture. The discrepancy may be due to differences in the populations studied; Høidrup et al. studied Danish adults and Mukamal studied US adults over 65 from four communities. Another possible reason for the discrepancy may lie in how the exposure variable was defined. Whereas Høidrup et al. modeled alcohol preference, Mukamal et al. modeled consumption of type using indicator variables. Our study seeks to reconcile inconsistencies in past studies of alcohol preference and to take past drinkers and non-drinkers into account. We will investigate the relationship between type of alcohol consumed and the risk of hip fracture in a large, ethnically and geographically diverse cohort of postmenopausal women in the United States with available data on potentially important confounders of the relationship including physical activity and falls.

## Methods

The question of interest was addressed using a large multi-ethnic cohort of postmenopausal women enrolled in either the Women’s Health Initiative (WHI) Clinical Trials (CT, N=68,133) or the Observational Study (OS, N=93,676) at 40 clinical centers across the United States between 1993 and 1998. The CT enrolled participants into one or more clinical trial components: the Hormone Therapy Trials, Dietary Modification Trial, or Calcium/Vitamin D Trial; those ineligible or unwilling to participate in the CT were invited to enroll in the OS, which also enrolled participants directly [29]. Closeout occurred from 2004–2005 for the main study. Study design and eligibility have been described previously [30]. The analysis was based on a cohort that included all eligible WHI participants in the CT and OS. The study was approved by Institutional Review Boards at each clinical center. All participants provided signed informed consent.

**Figure 1:**
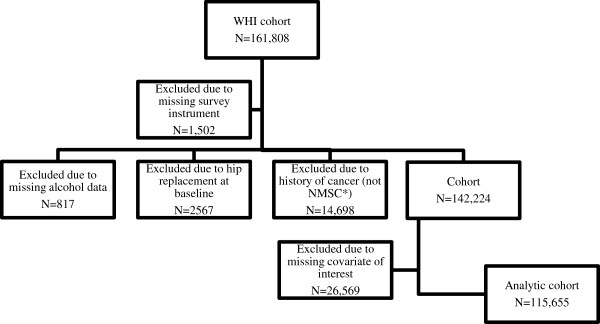
**Construction of the analytic cohort.** * NMSC: Non-melanoma skin cancer.

Figure 1 describes the derivation of the cohort. Eligible participants were those with data on alcohol consumption, with no history of cancer, and no evidence of hip replacement at baseline. Participants were considered ineligible if they were missing relevant data collection forms. There were 142,224 participants eligible for study. Due to missing covariates included in the scientific model, 26,569 were further excluded, yielding a final analytic data set of N=115,655 (15.2% missing).

Exclusions were largely due to missing data on prior hip fractures and parental hip fractures, both of which were not collected on forms for all participants. Underlying reasons for missing the latter, therefore, are unlikely to be related to participant characteristics. Some systematic differences were observed, however, between those participants who were excluded due to missing data (N=26,569) and those included in the analysis (N=115,655). For example, a higher proportion of white women were included in the analysis than among those excluded (83.65% versus 77.25%). Other differences included the percentage of falls (68.81% of those who were included reported no falls, compared to 61.29% of those who were excluded), and percentage of participants included in the OS cohort (57.70% compared to 45.77%). Rates of incident hip fracture, however, did not differ by inclusion status.

### Covariates

Type of alcohol consumed was measured in two ways. The first was a categorical variable that captured preference of type of alcohol. The second consisted of three non-mutually exclusive indicator variables for whether a specific type of alcohol was consumed, where the possibilities included beer, wine, and hard liquor. More specifically, for the former, seven mutually exclusive categories of alcohol preference were created; non-drinker, past drinker, and current drinkers who prefer beer, wine, hard liquor, have no strong preference, or drink very infrequently, as determined by food frequency questionnaire (FFQ) consumption patterns of beer, wine, and hard liquor at baseline. Current-beer was assigned to those whose beer consumption constituted 60 or more percent of their total alcohol consumption. Current-wine and current-hard liquor preference were defined similarly. Current-no strong preference was assigned to those who did not have a predominating type of alcohol. A participant was classified as current-infrequent if the participant reported being a current drinker but did not report consumption on their FFQ. The WHI FFQ was validated for alcohol consumption using four day food records and four day diet recalls [31].

### Hip fracture

The primary outcome was an indicator for having a hip fracture during the WHI study period. Hip fractures were centrally adjudicated at the WHI Bone Density Center by a physical adjudicator; participants reporting a hip fracture on the semi-annual (CT) or annual (OS) medical history update were contacted for additional information and medical records [32,33].

Adjustments were made for potential confounders as identified by prior analyses of hip fracture in the WHI and other cohorts as well as for clinically important variables. Demographic factors included race/ethnicity, age group and education at screening. Also included was a diagnosis of osteoporosis, bisphosphonate drug use, previous hip fracture at age 55 and up, and history of hip fracture at age 40 and up for either parent from participant medical history. Baseline risk factors included smoking status, hormone therapy use, physical activity in metabolic equivalents per week, BMI category as defined by WHO, and the number of falls the participant reported in the past year. All covariates were measured at baseline. We also included indicators for the relevant trial arms as defined by the WHI.

### Statistical analyses

The relationship between alcohol preference (modeled as a categorical variable) and risk of hip fracture was assessed using logistic regression methods. An alternative model that made use of indicators for each type of alcohol allowed investigation of associations between types of alcohol and risk of hip fracture as opposed to preference of alcohol type. Odds ratios that describe the association are presented after adjusting for listed confounders. Additionally, several covariates (falls in the past year, BMI, education and age) were explored as potential mediators and moderators of the relationship between alcohol consumption and hip fracture. Total alcohol consumption was also explored as a confounder.

As the CT and OS populations differ slightly, the two study cohorts were additionally analyzed separately as a sensitivity analysis. Data on red and white wine consumption were available in the OS cohort at year 1 of follow up; to explore differences between red and white wine those who preferred wine were additionally split based on whether they reported higher consumption of red wine, higher consumption of white wine, or equal consumption of red and white wine. Several additional sensitivity analyses were performed; in the first, participants reporting a prior hip fracture at baseline were excluded from analysis. The second excluded participants who reported having osteoporosis at baseline. A final sensitivity analysis, for the indicator model, defined alcohol use for moderate drinkers only (>1 drink/day) in accordance with the 2010 Dietary Guidelines for Americans [23]. All analyses were performed with SAS software, Version 9.3 (SAS Institute Inc., Cary, NC) of the SAS System for Windows.

## Results

**Figure 2:**
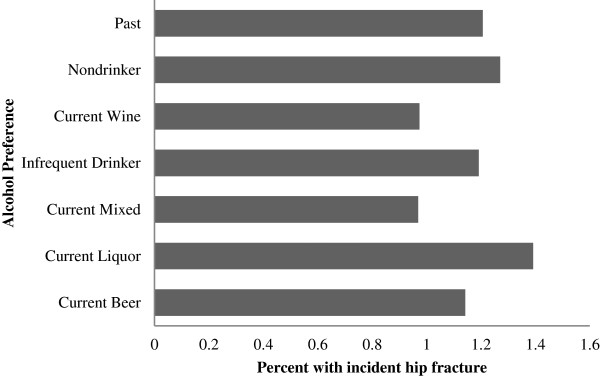
Percent of participants with incident hip fracture during the study period.

Table 1 presents baseline characteristics of the 115,655 women in the analytic cohort. On average they were 63.1 years old at study entry and 83.7% were white. Approximately 11.1% were non-drinkers, 18.0% were past drinkers and of the current drinkers, 47.4% preferred wine, 6.6% preferred beer, 12.7% preferred hard liquor, 15.7% had no strong preference, and 17.7% drank infrequently. Strong associations between baseline characteristics and preference of type of alcohol were observed. For example, 86.9% of non-drinkers also reported never having smoked whereas only 40.0% of the beer drinkers never smoked. In addition, wine drinkers reported more physical activity than any other category of alcohol preference. Only 1.3% of non-drinkers experienced a hip fracture during the follow up period, whereas percentages were even lower for all current alcohol consumer types with the exception of hard liquor drinkers (1.4%) and past consumers (1.2%) (Figure 2).

**Table 1:** Baseline demographic and risk factor characteristics by alcohol type preference in the WHI OS+CT cohort

**Covariate**	**Drinker type classification**							**Total**	**χ**^**2 **^**p-value**
	***Prefer beer***	***Prefer liquor***	***No preference***	***Infrequent drinker***	***Prefer wine***	***Non-drinker***	***Past drinker***		
Cohort	5405 4.7	10412 9.0	12876 11.1	14483 12.5	38841 33.6	12805 11.1	20833 18.0	115655	
**Characteristics at baseline**									
*Ethnicity*									<.0001
American Indian or Alaskan Native	29	33	42	53	100	78	116	451	
	0.5	0.3	0.3	0.4	0.3	0.6	0.6		
Asian or Pacific Islander	104	66	141	482	581	1253	660	3287	
	1.9	0.6	1.1	3.3	1.5	9.8	3.2		
Black or African-American	566	636	693	1256	1636	1702	3098	9587	
	10.5	6.1	5.4	8.7	4.2	13.3	14.9		
Hispanic/Latino	370	190	400	588	955	829	980	4312	
	6.9	1.8	3.1	4.1	2.5	6.5	4.7		
White (not of Hispanic origin)	4271	9407	11495	11923	35193	8741	15710	96740	
	79.0	90.4	89.3	82.3	90.6	68.3	75.4		
Other	65	80	105	181	376	202	269	1278	
	1.2	0.8	0.8	1.3	1.0	1.6	1.3		
*Age group at screening*									<.0001
<50-59	2251	2819	4981	5467	13379	3523	6667	39087	
	41.4	27.1	38.7	37.8	34.5	27.5	32.0		
60-69	2355	4917	5709	6324	17772	5896	9384	52357	
	43.6	47.2	44.3	43.7	45.8	46.0	45.0		
70-79+	799	2676	2186	2692	7690	3386	4782	24211	
	14.8	25.7	17.0	18.6	19.8	26.4	23.0		
*Education*									<.0001
High school or less	2038	3171	3234	5044	8858	5835	8625	36805	
	37.7	30.5	25.1	34.8	22.8	45.6	41.4		
Some college/AA	1410	3040	3575	4306	10568	3009	5708	31616	
	26.1	29.2	27.8	29.7	27.2	23.5	27.4		
College/post sec.	1957	4201	6067	5133	19415	3961	6500	47234	
	36.2	40.4	47.1	35.4	50.0	30.9	31.2		
*Osteoporosis*									<.0001
No	5104	9748	12148	13566	36291	11726	19075	107658	
	94.4	93.6	94.4	93.7	93.4	91.6	91.6		
Yes	301	664	728	917	2550	1079	1758	7997	
	5.6	6.4	5.7	6.3	6.6	8.4	8.4		
*Number of falls in the past 12 months*									<.0001
None	3727	7256	8670	9962	26733	9013	14223	79584	
	69.0	69.7	67.3	68.8	68.8	70.4	68.3		
1 time	1035	2009	2622	2830	7825	2340	3990	22651	
	19.2	19.3	20.4	19.5	20.2	18.3	19.2		
2 times	425	804	1104	1166	2973	956	1682	9110	
	7.9	7.7	8.6	8.1	7.7	7.5	8.1		
3 or more times	218	343	480	525	1310	496	938	4310	
	4.0	3.3	3.7	3.6	3.4	3.9	4.5		
*Prior hip fracture at baseline at age 55+*									0.0193
No	5391	10375	12849	14441	38723	12757	20744	115280	
	99.7	99.6	99.8	99.7	99.7	99.6	99.6		
Yes	14	37	27	42	118	48	89	375	
	0.3	0.4	0.2	0.3	0.3	0.4	0.4		
*Parent had hip fracture at age 55+*									<.0001
No	4763	8974	11148	12638	33545	11212	18303	100583	
	88.1	86.2	86.6	87.3	86.4	87.6	87.9		
Yes	642	1438	1728	1845	5296	1593	2530	15072	
	11.9	13.8	13.4	12.7	13.6	12.4	12.1		
*On bisphosphonate medication at screening*									<.0001
No	5328	10228	12670	14290	38020	12540	20481	113557	
	98.6	98.2	98.4	98.7	97.9	97.9	98.3		
Yes	77	184	206	193	821	265	352	2098	
	1.4	1.8	1.6	1.3	2.1	2.1	1.7		
*Amount of alcohol consumption*									NA
Servings per week	4.2	6.1	3.4	0	3.7	0	0	2.4	
	6.3	7.3	5.4	0.0	5.2	0.0	0.0	4.8	
*Category of alcohol consumption*									NA
Non-drinker	0	0	0	0	0	12805	0	12805	
	0.0	0.0	0.0	0.0	0.0	100.0	0.0		
Past drinker	0	0	0	0	0	0	20833	20833	
	0.0	0.0	0.0	0.0	0.0	0.0	100.0		
<1 drink per month	0	0	0	14483	0	0	0	14483	
	0.0	0.0	0.0	100.00	0.0	0.0	0.0		
<1 drink per week	1832	2373	5504	0	14135	0	0	23844	
	33.9	22.8	42.8	0.0	36.4	0.0	0.0		
1-<7 drinks per week	2580	4549	5702	0	17302	0	0	30133	
	47.7	43.7	44.3	0.0	44.6	0.0	0.0		
7+ drinks per week	993	3490	1670	0	7404	0	0	13557	
	18.4	33.5	13.0	0.0	19.1	0.0	0.0		
*Smoking status*									<.0001
Never Smoked	2161	3677	5729	8225	18982	11129	9938	59841	
	40.0	35.3	44.5	56.8	48.9	86.9	47.7		
Past Smoker	2623	5306	6284	5119	18011	1419	9370	48132	
	48.5	51.0	48.8	35.3	46.4	11.1	45.0		
Current Smoker	621	1429	863	1139	1848	257	1525	7682	
	11.5	13.7	6.7	7.9	4.8	2.0	7.3		
*HT Use*									<.0001
Never used	2451	4466	5018	6543	14962	6319	9666	49425	
	45.4	42.9	39.0	45.2	38.5	49.4	46.4		
Past user	790	1661	1839	2158	5609	1968	3489	17514	
	14.6	16.0	14.3	14.9	14.4	15.4	16.8		
Current user	2164	4285	6019	5782	18270	4518	7678	48716	
	40.0	41.2	46.8	39.9	47.0	35.3	36.9		
*Physical activity*									<.0001
MET hours per week	12.2	12.6	13.9	10.6	14.7	10.1	10.9	12.6	
	14.1	13.5	14.0	12.5	14.3	12.6	13.5	13.8	
*BMI*									<.0001
Underweight	54	69	71	85	353	168	201	1001	
	1.0	0.7	0.6	0.6	0.9	1.3	1.0		
Normal	1896	3711	4626	3989	16488	3965	5795	40470	
	35.1	35.6	35.9	27.5	42.5	31.0	27.8		
Overweight	1869	3816	4710	4845	13814	4278	6817	40149	
	34.6	36.7	36.6	33.5	35.6	33.4	32.7		
Obese I	1037	1797	2243	3256	5573	2603	4537	21046	
	19.2	17.3	17.4	22.5	14.4	20.3	21.8		
Obese II	365	691	834	1455	2952	1134	2131	8541	
	6.8	6.6	6.5	10.1	4.7	8.9	10.2		
Obese III	184	328	392	853	772	657	1352	4538	
	3.4	3.2	3.0	5.9	2.0	5.1	6.5		
*Incident hip fracture during follow up*									0.0065
No	5344	10272	12749	14308	38459	12640	20593	114365	
	98.9	98.7	99.0	98.8	99.0	98.7	98.9		
Yes	61	140	127	175	382	165	240	1290	
	1.1	1.3	1.0	1.2	1.0	1.3	1.2		
*Death during follow up*									<.0001
No	5127	9772	12357	13765	37194	12065	19323	109603	
	94.9	93.9	96.0	95.0	95.8	94.2	92.8		
Yes	278	640	519	718	1647	740	1510	6052	
	5.1	6.2	4.0	5.0	4.2	5.8	7.3		

Note: counts and percentages are presented for categorical variables; means and standard deviations are presented for continuous variables.

Consumption of alcohol was comparable across type of preference and relatively low (mean: 3.34 servings per week, SD: 5.46 among those reporting current alcohol consumption). Of those who prefer beer, average consumption was half a medium serving per day; this was also true for those who prefer wine. Those with a preference for hard liquor consumed three-fourths of a medium serving each day on average.

Preference of type of alcohol at baseline was strongly associated with risk of hip fracture (p=0.0167). Preferring wine was associated with 22% fewer hip fractures compared to non-drinkers; no other category was significantly different from non-drinkers. Further, wine drinkers experienced a reduced risk of hip fracture compared to beer drinkers (OR=0.72; 95% CI 0.55-0.95), hard liquor drinkers (OR=0.87; 95% CI 0.71-1.06), those with no strong preference (OR=0.89; 95% CI 0.73-1.10), infrequent drinkers (OR=0.73; 95% CI 0.61-0.88), and past consumers (OR=0.85; 95% CI 0.72-1.00). Asian, Black, and Hispanic women had a lower risk of hip fracture compared to white women; increasing age greatly increased the risk of hip fracture. Other risk factors for hip fracture included having osteoporosis, increasing number of falls in the past year, lower BMI category, less physical activity, current smoking, never using hormone therapy (HT), having a previous hip fracture at age 55+, and having a parent who fractured her or his hip at age 40+ (Table 2). When categories of current drinkers were combined, current drinkers had a 12.2% lower risk of hip compared to non-drinkers (p-value = 0.3510, not shown).

**Table 2:** Associations between alcohol type preference and incident hip fracture in the WHI OS+CT cohort

**Covariate**	**Unadjusted model**		**Adjusted model**	
	**Odds ratio (95% CI)**	**P-value**	**Odds ratio (95% CI)**	**P-value**
*Alcohol type preference*		p=0.0067		p=0.0167
Prefer beer vs. Non-drinker	0.87 (0.65, 1.18)		1.07 (0.79, 1.46)	
Prefer liquor vs. Non-drinker	1.04 (0.83, 1.31)		0.90 (0.71, 1.14)	
No strong preference vs. Non-drinker	0.76 (0.61, 0.96)		0.87 (0.68, 1.11)	
Infrequent drinker vs. Non-drinker	0.94 (0.76, 1.16)		1.06 (0.85, 1.32)	
Prefer wine vs. Non-drinker	0.76 (0.63, 0.91)		0.78 (0.64, 0.95)	
Past drinker vs. Non-drinker	0.89 (0.73, 1.09)		0.92 (0.75, 1.13)	
*Ethnicity*				p<.0001
American Indian or Alaska Native vs. White			0.81 (0.30, 2.18)	
Asian or Pacific Islander vs. White			0.32 (0.19, 0.53)	
Black or African-American vs. White			0.32 (0.22, 0.46)	
Hispanic or Latino vs. White			0.31 (0.17, 0.55)	
Other vs. White			0.51 (0.25, 1.02)	
*Age group at screening*				p<.0001
60-69 vs. 50-59			2.78 (2.25, 3.45)	
70-79 vs. 50-59			9.37 (7.59, 11.56)	
*Education*				p=0.8491
HS vs. College			1.04 (0.91, 1.19)	
Some college vs. College			1.03 (0.90, 1.19)	
*Osteoporosis at screening*				p<.0001
No vs. Yes			0.58 (0.49, 0.68)	
*Falls in the past year*				p<.0001
1 vs. None			1.15 (1.00, 1.32)	
2 vs. None			1.45 (1.20, 1.75)	
3+ vs. None			2.13 (1.69, 2.68)	
*Previous hip fracture at age 55+*				p=0.0005
No vs. Yes			0.45 (0.28, 0.70)	
*Parent had hip fracture at age 40+*				p<.0001
No vs. Yes			0.68 (0.59, 0.78)	
*Bisphosphonate drug at screening*				p=0.1166
No vs. Yes			1.30 (0.94, 1.81)	
*Smoking status*				p=0.0053
Current vs. Never			1.42 (1.15, 1.77)	
Past vs. Never			1.02 (0.91, 1.16)	
*Hormone therapy status*				p<.0001
Current vs. Never			0.65 (0.57, 0.74)	
Past vs. Never			0.75 (0.64, 0.87)	
*Physical activity*				p<.0001
METs per week			0.99 (0.98, 0.99)	
*BMI Category*				p<.0001
Underweight vs. Normal			1.98 (1.37, 2.85)	
Overweight vs. Normal			0.67 (0.58, 0.76)	
Obese I vs. Normal			0.49 (0.41, 0.58)	
Obese II vs. Normal			0.52 (0.40, 0.68)	
Obese III vs. Normal			0.39 (0.25, 0.59)	

Table 2 is adjusted for alcohol type preference, ethnicity, age group, education, osteoporosis, falls in the past year, bisphosphonate drug use, smoking status, HT status, physical activity measured in MET hours, BMI category, HT trial arm, CaD trial arm, OS versus CT cohort, previous hip fracture at age 55+, and parental history of hip fracture at age 40+.

To evaluate whether specific types of alcohol consumed (beer, wine or hard liquor) are associated with risk of hip fracture, indicator values were incorporated. Specifically, indicators for consuming at least one serving of beer per week, consuming at least one serving of wine per week, and consuming at least one serving of hard liquor per week were jointly included in the model. Whereas no association was observed between beer consumption or hard liquor consumption and risk of hip fracture, a protective association was observed for wine, where the odds ratio of 0.75 (95% CI 0.64-0.87) suggested that wine drinkers have a 25% reduction in the risk of hip fracture after adjusting for consumption of other alcohol types and other confounders (Table 3).

**Table 3:** Associations between consumption of beer, wine and liquor and incident hip fracture in the WHI CT+OS cohort

	**Logistic model with alcohol consumption indicators**			
**Model**	**HR (95% CI)**	**P-value**	**HR (95% CI)**	**P-value**
*Consumes one or more servings of beer per week*		p=0.8756		p=0.3267
Beer, yes vs. no	0.98 (0.75, 1.28)		1.14 (0.88, 1.49)	
*Consumes one or more servings of liquor per week*		p=0.0004		p=0.4408
Liquor, yes vs. no	1.37 (1.15, 1.63)		1.07 (0.90, 1.28)	
*Consumes one or more servings of wine per week*		p=0.0010		p=0.0002
Wine, yes vs. no	0.78 (0.67, 0.90)		0.75 (0.64, 0.87)	

Table 3 is adjusted for beer consumption, liquor consumption, wine consumption, ethnicity, age group, education, osteoporosis, falls in the past year, bisphosphonate drug use, smoking status, HT status, physical activity measured in MET hours, BMI category, HT trial arm, CaD trial arm, OS versus CT cohort, previous hip fracture at age 55+, and parental history of hip fracture at age 40+.

To investigate the potential effect of amount of alcohol consumed, total alcohol servings per week was explored as a confounder both categorically and continuously. Total amount consumed was not significantly associated with hip fracture (category: p=0.6827, amount: p=0.8162), nor did it affect the coefficients corresponding to alcohol preference. Due to the low consumption in this cohort, the U-shaped relationship with hip fracture was not observed.

Age at menopause and dietary covariates for fruit, vegetable, fiber and dairy consumption were not included in the final models, as they did not change the coefficients corresponding to the covariate of interest and were not associated with hip fracture in exploratory analyses. The number of falls in the past year was explored as a potential moderator. A test for an interaction effect between falls and alcohol preference was not rejected (p-value=0.5846), suggesting that falls do not moderate the relationship between alcohol preference and hip fracture. Falls were also explored as a potential mediator; models fit with and without falls did not attenuate the effect of alcohol preference or change the point estimates for alcohol preference in a meaningful way. Additionally, there was not sufficient evidence to suggest the BMI modified the association between type of preference and hip fracture (p=0.7218). Interactions between preference and education (p=0.2301) and preference and age group (p=0.4120) were similarly not significant. We did not find evidence of effect modification by study cohort (CT and OS); further, when analyses were stratified by cohort, associations were in the same direction. Among the participants in the OS, those who preferred white wine, red wine, or both types had a lower risk of hip fracture compared to non-drinkers, however, this association was not significant (p=0.8731). In addition, indicators for more than one serving of red wine per week and more than one serving of white wine per week was not significant (p=0.6246 and p=0.1124 respectively).

### Sensitivity analyses

To assess the influence of women with a prior hip fracture at baseline after age 55 on our findings, the analysis was repeated after excluding these women. The association of interest remained (p=0.0112, OR for wine preference compared to non-drinkers = 0.76; 95% CI 0.62-0.92). Results were similar for the second sensitivity analysis, in which participants who reported having osteoporosis at baseline were excluded (p=0.0071, OR for wine preference compared to non-drinkers = 0.74; 95% CI 0.60-0.92). When indicators for beer, wine and liquor consumption were defined as more than 7 drinks per week in accordance with the 2010 Dietary Guidelines for Americans definition of moderate drinking, point estimates were in the same direction as the analysis defining consumption as more than 1 drink per week.

## Discussion

We found compelling variation in risk of hip fracture by type of alcohol preference, where women who preferred wine appeared to have the lowest risk of hip fracture. In an alternative model, where the association of each type of alcohol consumed on risk of hip fracture was explored, we found that wine drinkers had a lower risk of hip fracture relative to those who did not report consuming wine at baseline.

We are not the first to investigate this question. In a Danish cohort consisting of 31,785 participants, Høidrup et al. found a significantly protective association with wine preference in age-, sex- and study-adjusted models, however the association did not persist after adjusting for BMI, smoking, physical activity, and education [19]. While our conclusions are similar, our study also found that the relationship between preferring wine compared to non-drinking and hip fracture was significant in a model adjusted for the same covariates and other important predictors of hip fracture including falls in the past year and history of hip fracture. Our results, however, are generalizable only to postmenopausal women and our cohort on average reported less alcohol consumption than the Danish cohort investigated by Høidrup.

Although their primary objective was assessing amount of alcohol consumed, Mukamal et al. also examined beer, wine, and liquor consumption in 5,865 participants in the Cardiovascular Health Study. They included 0 (reference), <1, 1–6, and 7+ drinks per week categories for beer, wine, and liquor and noted that no type was significantly associated with hip fracture [20]. The HR for 1–6 drinks per week of wine was 0.75 (95% CI 0.48-1.17), which is similar to our finding that consumption of at least one serving of wine per week is associated with a reduction in risk of hip fracture of 25%.

Previous studies have suggested that moderate alcohol consumption is protective in terms of hip fracture. For example, although they did not distinguish between type of alcohol consumed, Marrone et al. found a positive relationship between alcohol consumption and bone mineral density (BMD) of the hip in a cohort of postmenopausal women. They also found increased bone turnover in non-drinkers compared to those who drank alcohol [34].

Our analysis suggests that much of the relationship between alcohol and hip fracture may be due to wine consumption. There are likely to be many unidentified differences in individuals who prefer wine, beer or spirits. Mortensen et al. identified some of these factors [35].

Because of the observational nature of this analysis we can only speculate on the mechanisms of the associations we found. One possible explanation for this association is the resveratrol content in wine. In a study of bone loss in tail-suspended rats, resveratrol protected against bone loss during immobilization [36]. Resveratrol also acts as an inhibitor of adipogenesis and may potentially promote osteoblast formation [37].

Hip and other osteoporotic fractures often result from falls [2] and alcohol intake has been shown to influence falls [20,22]. The J-shaped pattern observed between amount of alcohol consumption and falls is similar to that observed with hip fractures [38], suggesting that falls may act as a mediator. We, however, found no evidence of an interaction between falls in the past year at baseline and alcohol preference on incident hip fracture; this suggests that the association of alcohol preference does not vary between those who have and have not experienced falls. Furthermore, by comparing coefficients for preference of type of alcohol in models with and without an indicator for falling we examined whether falling is a possible pathway by which alcohol may affect hip fracture. As no attenuation of the association was observed between models, there was not sufficient evidence to indicate this as the case.

This analysis has a number of strengths; data are from a large ethnically and racially diverse cohort of postmenopausal women and contained a large number of hip fracture events. The outcome of interest, hip fracture, was centrally adjudicated by the WHI Clinical Coordinating Center. Further, the WHI collected many variables of interest in assessing hip fracture risk including falls, previous hip fractures, and family history of hip fracture that have not been fully evaluated in previous research.

**Figure 3:**
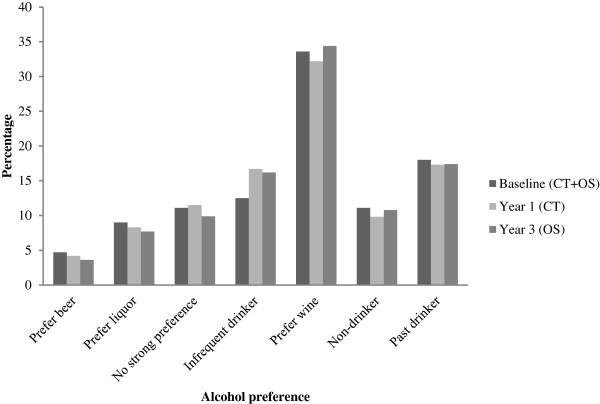
Alcohol preference at baseline (entire cohort, CT+OS), year 1 (CT only), and year 3 (OS only).

There are several limitations as well. Preference of alcohol type was defined at baseline for each participant and does not capture lifetime consumption pattern, however, data from follow up assessments of beer, wine and hard liquor consumption suggest that preference does not change greatly (Figure 3).

Further, we are unable to classify past drinkers by type preference due to the lack of historical alcohol consumption data. Information on past alcohol preference would have allowed us to assess the effect of past preferences along with current preferences, which is of interest as alcohol preference tends to differ greatly for younger and older adults [39]. Also, preference of alcohol type was assigned based on reported alcohol consumption and on the food frequency questionnaire. However, in a subset of women in the WHI, FFQs were compared with four-day food records and 24-hour recalls; correlation between FFQ alcohol consumption and eight days of intake (four from recall, four from food records) was 0.86 for alcohol. The intra-class correlation coefficient for test-retest reliability for alcohol was 0.92, suggesting that for this cohort alcohol consumption is adequately captured by FFQ [31].

Interestingly, total alcohol servings was explored as a confounder and not found to alter results. A possible explanation is that the overall variability for total alcohol consumed was low. Lack of variability in total amount consumed and low median alcohol intake in this cohort may affect the generalizability of our results. Also, as the FFQ did not ask participants the amounts of red and white wine consumed, we were unable to differentiate wine drinkers into red wine drinkers and white wine drinkers except in the OS cohort.

Our analysis did not account for varying lengths of follow up time. We also considered and fit Cox proportional hazards models, using adjudicated time to hip fracture and censored at the end of the main study, death, or last follow up visit. Findings were comparable based on this model. As hip fracture is a rare event (<2% in the cohort during the study period) and follow-up time is comparable among women across the levels of the preference variable, our model choice of a logistic regression was justified.

Unmeasured confounders of the relationship between hip fracture and alcohol preference may exist. Although we attempted to account for socio-demographic covariates and dietary preferences by adjusting for education in the model, wine drinkers may differ by other endogenous characteristics or lifestyle factors that we did not observe. Further, other socioeconomic characteristics such as income [40,41], type of housing [40,42], living with someone [43] or deprivation [44] might have been important confounders even after adjusting for education. These characteristics are likely to be related to alcohol preference especially with regards to wine [35]. Finally, not having BMD measurements on this population limited us from investigating the role of alcohol type on bone density, one important mechanism to consider.

## Conclusions

Postmenopausal women who are current alcohol consumers and prefer wine experience significantly fewer hip fractures compared to women who are non-drinkers, as well as those who prefer beer, those who prefer liquor, those with no strong preference, those who drink infrequently, and those who report being past alcohol consumers. The variability in risk of hip fracture by type of alcohol appears to be driven by the protective association seen here for wine drinkers. Note that these results generalize to a cohort of older women who are largely light drinkers and may not apply to men or moderate to heavy drinkers.

## Abbreviations

WHI: Women’s Health Initiative; CT: Clinical Trial; OS: Observational Study; FFQ: Food Frequency Questionnaire; BMD: Bone mineral density; HT: Hormone Therapy.

## Competing interests

The authors have no conflicts of interest to report.

## Authors’ contributions

All authors contributed to this work. Specifically, JTK participated in designing the research plan, performed all statistical analyses and co-wrote the manuscript. MLS, JR, JWW, MRC and MF participated in designing the research plan, provided feedback on analyses, and edited the manuscript. MD participated in developing the research plan, directed the analyses and co-wrote the manuscript. All authors read and approved the final version of the manuscript for submission.

## Pre-publication history

The pre-publication history for this paper can be accessed here:

http://www.biomedcentral.com/1472-6874/13/36/prepub
